# Risk of coronary heart disease among cancer survivors with different prediagnosis body mass index

**DOI:** 10.1038/s41598-021-82026-5

**Published:** 2021-01-28

**Authors:** Ahryoung Ko, Kyuwoong Kim, Joung Sik Son, Yu Jin Cho, Sang Min Park, Minseon Park

**Affiliations:** 1 Department of Public Health and Medical Service, Seoul National University Hospital, Seoul, Republic of Korea; 2 Department of Clinical Medical Sciences, College of Medicine, Seoul National University, Seoul, Republic of Korea; 3 Division of Cancer Control and Policy, National Cancer Control Institute, National Cancer Center, Goyang, Republic of Korea; 4 Department of Biomedical Sciences, Seoul National University Graduate School, Seoul, Republic of Korea; 5 Department of Family Medicine, Seoul National University Hospital, Seoul, Republic of Korea; 6 Department of Family Medicine, College of Medicine, Seoul National University, 101 Daehak-ro, Jongno-gu, Seoul, Republic of Korea

**Keywords:** Cancer epidemiology, Cancer, Health care, Risk factors

## Abstract

Association between body mass index (BMI) and coronary heart disease (CHD) in cancer survivors is not clearly established. This study analyzed the prediagnosis BMI-CHD association by examining 13,500 cancer survivors identified from the National Health Insurance Service-Health Screening Cohort from January 1, 2004 to December 31, 2009 including the patients who were free of cardiovascular disease at enrollment. The Cox proportional hazards model (adjusted for socioeconomic, health behavior, health status, and medical characteristics) was used for calculating hazard ratios (HR) and 95% confidence intervals (95% CI) for CHD in each prediagnosis BMI category among cancer survivors. Compared to cancer survivors with a prediagnosis BMI between 18.5 and 22.9 kg/m^2^, those with a prediagnosis BMI of 23.0–24.9 kg/m^2^ and ≥ 25.0 kg/m^2^ had significantly higher CHD risk (HR = 1.51; 95% CI: 1.13–2.01 and HR = 1.38; 95% CI: 1.04–1.84, respectively). Cancer survivors with a low prediagnosis BMI (< 18.5 kg/m^2^) also had significantly higher CHD risk (HR = 1.97; 95% CI: 1.20–3.24) compared to those with a BMI of 18.5–22.9 kg/m^2^. Similar associations were found after stratifying analyses based on first cancer site and sociodemographic and medical characteristic subgroups. Our study suggests that prediagnosis underweight among patients with cancer is a predictor of CHD risk.

## Introduction

As obesity has become a worldwide epidemic, a potential increase in obesity-related morbidity and mortality has become a major issue in the public medicine field^1,2^. In both clinical studies and daily practice, body mass index (BMI) is usually utilized for measuring the degree of obesity^3,4^. There is a general consensus regarding the positive association between higher BMI levels and the increasing incidence of cardiovascular disease (CVD) in the general population^5^. However, recently, cardiovascular and metabolic research has presented extensive evidence for a U- or J-shaped relationship between mortality and BMI, which poses a significant challenge for this obesity-disease paradigm^6,7^. Recent studies have presented evidence of an “obesity paradox,” wherein lower BMI is a risk factor for increased mortality^8–10^. Several reports have also associated morbidity with underweight; for example, lower BMI has been correlated with increased coronary heart disease (CHD) risk in the general population. An Indonesian cross-sectional field study showed that underweight was an important risk factor for CHD. Based on nationwide health-related telephone surveys in the USA, Park and colleagues also suggested that underweight may be an independent risk factor for CHD in the general population^11^. Increased CHD risk in the underweight general population could be associated with various clinical factors, such as low cardiorespiratory fitness (CRF)^6^, metabolically unhealthy status^12,13^, and body fat distribution^14^, which are particularly relevant to underweight cancer survivors.

Therapeutic advances, despite improving longevity, have increased the overlap between various diseases, with millions of cancer survivors now at risk of developing CHD. Previous studies have examined the multiple inherent risks in the specific outcomes of CHD in survivors of varied site-specific cancers^7,15,16^. As few studies have investigated underweight patients with cancer as an independent group in CHD risk assessment, this study aimed to examine the impact of prediagnosis underweight by using data from a previous population-based longitudinal study on CHD risk.

## Results

### Characteristics of cancer survivors based on prediagnosis BMI (Table 1)

**Table 1:** Baseline characteristics of cancer survivors at enrollment according to body mass index (BMI) categories in the National Health Insurance-Health Screening cohort (NHIS-HEALS) database.

	Prediagnosis BMI (kg/m^2^)					
	< 18.5 (n = 621)	18.5–22.9 (n = 5305)	23.0–24.9 (n = 3392)	≥ 25.0 (n = 4182)	Total (n = 13,500)	*p* value^g^
Age, mean (SD)	67.5 (9.55)	63.1 (9.94)	61.9 (9.61)	60.7 (9.35)	62.3 (9.78)	< 0.001
**Sex**						
Male	494 (79.6)	3872 (73.0)	2365 (69.7)	2696 (64.5)	9427 (69.8)	< 0.001
Female	127 (20.4)	1433 (27.0)	1027 (30.3)	1486 (35.5)	4073 (30.2)	
Residential area						
Capital	73 (11.8)	783 (14.8)	569 (16.8)	700 (16.7)	2125 (15.7)	< 0.001
Metropolitan	222 (35.8)	2143 (40.4)	1434 (42.3)	1812 (43.3)	5611 (41.6)	
City/Town	326 (52.4)	2379 (44.8)	1389 (40.9)	1670 (40.0)	5764 (42.7)	
**Insurance premium**^a^						
1Q	115 (18.5)	954 (17.9)	514 (15.2)	631 (15.1)	2214 (16.4)	< 0.001
2Q	176 (28.3)	1173 (22.1)	702 (20.7)	858 (20.5)	2909 (21.5)	
3Q	175 (28.2)	1542 (29.1)	978 (28.8)	1267 (30.3)	3962 (29.4)	
4Q	155 (25.0)	1636 (30.9)	1198 (35.3)	1426 (34.1)	4415 (32.7)	
**Cigarette smoking**						
Non-smoker	305 (49.1)	3004 (56.6)	2065 (60.9)	2780 (66.5)	8154 (60.4)	< 0.001
Past smoker	58 (9.3)	513 (9.7)	417 (12.3)	478 (11.4)	1466 (10.9)	
Current smoker	258 (41.6)	1788 (33.7)	910 (26.8)	924 (22.1)	3880 (28.7)	
**Alcohol consumption**						
1–2 times/week	421 (67.8)	3564 (67.2)	2332 (68.8)	2945 (70.4)	9262 (68.6)	< 0.001
3–4 times/week	116 (18.7)	1197 (22.6)	803 (23.7)	998 (23.9)	3114 (23.1)	
≥ 5 times/week	84 (7.5)	544 (10.2)	257 (7.5)	239 (5.7)	1124 (8.3)	
**Physical activity**						
1–2 times/week	526 (84.7)	4339 (81.8)	2661 (78.5)	3238 (77.4)	10,764 (79.7)	< 0.001
3–4 times/week	47 (7.6)	562 (10.6)	435 (12.8)	571 (13.7)	1615 (12.0)	
≥ 5 times/week	48 (7.7)	404 (7.6)	296 (8.7)	373 (8.9)	1121 (8.3)	
Fasting serum glucose, mg/dL, mean, mean (SD)	103.0 (51.3)	101.9 (38.3)	103.3 (36.1)	105.3 (37.4)	103.3 (38.2)	< 0.001
Total cholesterol, mg/dL, mean, mean (SD)	174.3 (37.8)	186.8 (38.6)	195.3 (39.2)	199.1 (40.0)	192.2 (39.7)	< 0.001
**Blood pressure, mmHg** **mean, mean (SD)**						
SBP	123.1 (19.0)	126.6 (17.8)	129.7 (17.7)	131.7 (17.3)	128.8 (17.8)	< 0.001
DBP	75.8 (11.6)	77.9 (10.9)	80.1 (11.2)	81.5 (11.2)	79.5 (11.3)	< 0.001
**Charlson comorbidity index**						
0	103 (16.6)	1014 (19.1)	649 (19.2)	801(19.2)	2567 (19.0)	0.479
1	205 (33.1)	1859 (35.1)	1176 (34.7)	1428 (34.2)	4668 (34.6)	
≥ 2	311 (50.4)	2424 (45.8)	1563 (46.1)	1951 (46.6)	6249 (46.3)	
**ESC SCORE**^b^						
Very high (≥ 10%)	27 (4.3)	240 (4.5)	202 (5.9)	256 (6.1)	1328 (9.8)	< 0.001
High (5% ≤ SCORE < 10%)	418 (67.3)	2599 (49.0)	1481 (43.7)	1639 (39.2)	5310 (39.3)	
Moderate (1% ≤ SCORE < 5%)	144 (23.2)	1917 (36.1)	1369 (40.4)	1880 (44.9)	6137 (45.5)	
Low (SCORE < 1%)	32 (5.2)	549 (10.4)	340 (10.0)	407 (9.8)	725 (5.4)	
Family history of CVD^c^	6 (1.1)	79 (1.6)	58 (1.9)	72 (1.9)	215 (1.8)	< 0.001
Presence of depression^d^	40 (6.4)	301 (5.7)	198 (5.8)	192 (4.6)	731 (5.4)	< 0.001
**Medication use**^e^						
Aspirin	147 (23.7)	1409 (26.6)	1166 (34.4)	1718 (41.1)	1052 (7.8)	< 0.001
Statin	37 (6.0)	432 (8.1)	384 (11.3)	612 (14.6)	1465 (10.9)	< 0.001
Anti-hypertensive drugs	17 (2.7)	286 (5.4)	278 (8.2)	471 (11.7)	4815 (35.7)	< 0.001
Anti-diabetic drugs	51 (8.2)	517 (9.8)	424 (12.5)	625 (15.0)	1617 (12.0)	< 0.001
NSAIDs	215 (34.6)	1839 (34.7)	1187 (35.0)	1574 (37.6)	4440 (32.9)	< 0.001
**First site of cancer at enrollment**						
Lung cancer	152 (24.5)	987 (18.6)	483 (14.2)	505 (12.1)	2127 (15.8)	< 0.001
Stomach cancer	184 (29.6)	1334 (25.2)	813 (24.0)	1032 (24.7)	3363 (24.9)	< 0.001
Colorectal cancer	88 (14.2)	890 (16.8)	621 (18.3)	813 (19.4)	2412 (17.9)	< 0.001
Liver cancer	69 (11.1)	770 (14.5)	498 (14.7)	664 (15.9)	2001 (14.8)	< 0.001
Others^f^	184 (29.6)	1818 (34.3)	1233 (36.4)	1469 (35.1)	4704 (34.8)	< 0.001

Data above are presented in n(%) unless otherwise stated.

*Q* quartile, *sd* standard deviation, *ESC* European Society of Cardiology, *SCORE* systematic coronary risk evaluation, *CVD* cardiovascular disease, *NSAIDs* non-steroidal anti-inflammatory drugs, *ANOVA* analysis of variance.

^a^Proxy for income status (does not necessary represent the exact economic status).

^b^Based on the ESC SCORE chart for low risk countries.

^c^Family history of heart disease or stroke, based on a self-reported questionnaire.

^d^Based on the outpatient visit or hospital admission records with claims code for major depressive disorder/major depressive disorder, recurrent (ICD-10 codes: F32 and F33).

^e^Based on the prescription data for the patients in the NHIS-HealS database.

^f^Excludes thyroid cancer and other first site cancers listed in the cateogry.

^g^*p*-value from chi-square test for categorical variables and ANOVA for continuous variables for BMI groups.

Cancer survivors in the underweight category (prediagnosis BMI: < 18.5 kg/m^2^) had the highest mean age during enrollment (Table 1). Cancer survivor patients included in the final analytic sample were predominantly male (69.8%). Residential area and insurance premium distribution was not significantly different based on prediagnosis BMI. However, cancer survivors in the underweight category had the highest cigarette smoking rate (41.6%). Cancer survivors with higher prediagnosis BMI tended to have higher total cholesterol and blood pressure levels. The Charlson comorbidity index values were similar across the prediagnosis BMI categories. Cancer survivors with a prediagnosis BMI of ≥ 25.0 kg/m^2^ had the highest proportion of very high European Society of Cardiology Systematic Coronary Risk Evaluation (ESC SCORE) values (6.1%), whereas those with a prediagnosis BMI of < 18.5 kg/m^2^ had the lowest proportion (4.3%).

### Association between prediagnosis BMI and CHD (Table 2)

**Table 2:** Hazard ratio (HR) and 95% confidence (95% CI) intervals for coronary heart disease (CHD) in cancer survivors stratified by prediagnosis body mass index (BMI) categories.

	Pre-diagnosis BMI (kg/m^2^)			
	< 18.5 (n = 621)	18.5–22.9 (n = 5305)	23.0–24.9 (n = 3392)	≥ 25.0 (n = 4182)
**Coronary heart disease**				
Cases per 1000 person-years	9.14	4.04	6.02	5.53
Adjusted HR (95% CI), Model 1^a^	1.88 (1.16–3.04)**	1 (reference)	1.59 (1.22–2.08)***	1.55 (1.19–2.00)**
Adjusted HR (95% CI), Model 2^b^	1.96 (1.19–3.22)**	1 (reference)	1.56 (1.17–2.09)**	1.50 (1.01–1.94)**
Adjusted HR (95% CI), Model 3^c^	1.97 (1.20–3.24)**	1 (reference)	1.51 (1.13–2.01)**	1.38 (1.04–1.84)*

Data above are presented in numbers, HR, and HR (95% CI).

*HR* hazard ratio, *CI* confidence interval, *BMI* body mass index, *CHD* coronary heart disease, *NSAIDs* non-steroidal anti-inflammatory-drugs, *ICD* international classification of diseases, *NC* not calculable.

NOTE: Coronary heart disease includes ICD-10 codes I20-I25.

^a^Calculated from Cox proportional hazards model adjusted for socioeconomic factors (age, sex, residential area, insurance premium).

^b^Adjusted for lifestyle (smoking status, alcohol consumption, and physical activity), medical characteristics (fasting serum glucose, total cholesterol, systolic blood pressure, and Charlson comorbidity index), and family history (cardiovascular disease) in addition to the variables included in Model 1.

^c^Adjusted for depression and medication use (aspirin, statin, anti-hypertensive drugs, anti-diabetic drugs, and NSAIDs).

Depression was defined as outpatient visit or hospital admission with claims code for major depression disorder/major depression disorder, recurrent (ICD-10 codes: F32 and F33).

*p < 0.05, **p < 0.01, ***p < 0.001.

During the 69,801 person-years of follow-up, 364 CHD cases occurred among 13,500 cancer survivors (Table 2). A multivariable-adjusted Cox proportional hazards model (Model 3) indicated that cancer survivors with a prediagnosis BMI of 23.0–24.9 kg/m^2^ and ≥ 25.0 kg/m^2^ had significantly higher CHD risks (HR = 1.51; 95% CI: 1.13–2.01 for a prediagnosis BMI of 23.0 to 24.9 kg/m^2^ and HR = 1.38; 95% CI: 1.04–1.84 for a prediagnosis BMI of ≥ 25.0 kg/m^2^) compared to those with a prediagnosis BMI of 18.5 to 22.9 kg/m^2^. Furthermore, a prediagnosis BMI of < 18.5 kg/m^2^ was significantly associated with increased CHD risk (HR = 1.97; 95% CI: 1.20–3.24) compared to a prediagnosis BMI of 18.5 to 22.9 kg/m^2^. Analyses with Model 1 and Model 2 generated similar results.

### CHD risk based on prediagnosis BMI by first site of cancer (Table 3)

**Table 3:** Hazard ratios for coronary heart disease (CHD) among cancer survivors according to prediagnosis body mass index (BMI) stratified by first site of cancer at enrollment.

	Prediagnosis BMI (kg/m^2^)			
	< 18.5	18.5–22.9	23.0–24.9	≥ 25.0
**Lung cancer survivors** (***N*** = **2127**)				
No. of CHD cases	6	16	10	16
Multivariable-adjusted HR (95% CI)^a^	3.08 (1.16–8.15)*	1 (reference)	1.13 (0.49–2.61)	1.68 (0.78–3.62)
**Stomach cancer survivors** (***N*** = **3363**)				
No. of CHD cases	8	26	35	32
Multivariable-adjusted HR (95% CI)^a^	2.99 (1.31–6.80)**	1 (reference)	1.87 (1.07–3.28)*	1.42 (0.80–2.52)
**Colorectal cancer survivors** (***N*** = **2412**)				
No. of CHD cases	2	32	26	28
Multivariable-adjusted HR (95% CI)^a^	NC	1 (reference)	1.01 (0.57–1.80)	0.83 (0.47–1.48)
**Liver cancer survivors** (***N*** = **2001**)				
No. of CHD cases	1	12	8	9
aHR (95% CI)^a^	NC	1 (reference)	1.14 (0.43–3.00)	1.01 (0.39–2.62)
**Other types** (***N*** = **4704**)				
No. of CHD cases	4	27	41	51
Multivariable-adjusted HR (95% CI)^a^	NC	1 (reference)	2.21 (1.33–3.68)**	2.07 (1.23–3.47)**

Data above are presented in numbers, HR, and HR (95% CI).

*HR* hazard ratio, *CI* confidence interval, *NSAIDs* non-steroidal anti-inflammatory-drugs, *NC* not calculable.

^a^Adjusted for socioeconomic factors (age, sex, residential area, insurance premium), lifestyle (smoking status, alcohol consumption, and physical activity), medical characteristics (fasting serum glucose, total cholesterol, blood pressure, and Charlson comorbidity index), family history (cardiovascular disease), depression, and medication use (aspirin, statin, anti-hypertensive drugs, anti-diabetic drugs, and NSAIDs).

*p < 0.05, **p < 0.01, ***p < 0.001.

When we categorized cancer survivors according to their first cancer site at enrollment, there were 2127 lung cancer survivors, 3363 stomach cancer survivors, 2412 colorectal cancer survivors, 2001 liver cancer survivors, and 4704 survivors of other types of cancer (Table 3). Among cancer survivors with a prediagnosis BMI of < 18.5 kg/m^2^ whose first cancer site was the lungs cancer or stomach cancer, the multivariable adjusted HRs and 95% CIs were 3.08 (1.16–8.15) and 2.99 (1.31–6.80), respectively. The number of CHD cases (less than five) among survivors of colorectal cancer, liver cancer, and other cancers was too low for any accurate analysis of any prediagnosis BMI-CHD associations.

### Subgroup analyses of prediagnosis BMI-CHD associations (Table 4)

**Table 4:** Subgroup analysis of coronary heart disease (CHD) risk among cancer survivors by prediagnosis body mass index (BMI).

	Prediagnosis BMI (kg/m^2^)			
	< 18.5	18.5–22.9	23.0–24.9	≥ 25.0
**Age at first cancer diagnosis**				
< 60 years, No of CHD	5	36	32	38
Multivariable-adjusted HR (95% CI)^a^	2.00 (0.69–5.86)	1 (reference)	1.15 (0.70–1.91)	0.72 (0.43–1.22)
≥ 60 years, No of CHD	15	69	77	92
Multivariable-adjusted HR (95% CI)^a^	2.22 (1.26–3.93)**	1 (reference)	1.67 (1.17–2.37)**	1.75 (1.24–2.47)**
**Sex**				
Male, No of CHD	18	87	83	77
Multivariable-adjusted HR (95% CI)^a^	2.11 (1.24–3.58)**	1 (reference)	1.40 (1.01–1.93)*	1.15 (0.82–1.62)
Female, No of CHD	2	18	26	53
Multivariable-adjusted HR (95% CI)^a^	NC	1 (reference)	2.07 (1.08–3.98)*	2.43 (1.33–4.43)**
**Insurance premium**				
Upper Half, No of CHD	8	60	71	82
Multivariable-adjusted HR (95% CI)^a^	1.62 (0.76–3.45)	1 (reference)	1.54 (1.06–2.22)*	1.43 (0.99–2.05)
Lower Half, No of CHD	12	45	38	48
Multivariable-adjusted HR (95% CI)^a^	2.26 (1.15–4.44)*	1 (reference)	1.41 (0.88–2.26)	1.29 (0.80–2.06)
**Physical activity**				
< 3 times per week, No of CHD	18	98	102	117
Multivariable-adjusted HR (95% CI)^a^	1.89 (1.12–3.20)*	1 (reference)	1.55 (1.15–2.09)**	1.35 (0.99–1.82)
≥ 3 times per week, No of CHD	2	7	7	13
Multivariable-adjusted HR (95% CI)^a^	NC	1 (reference)	1.01 (0.32–3.26)	1.88 (0.71–4.99)
**Cigarette smoking**				
Non-smoker, No of CHD	5	46	62	90
Multivariable-adjusted HR (95% CI)^a^	1.22 (0.48–3.11)	1 (reference)	1.78 (1.19–2.66)**	1.76 (1.20–2.59)**
Past smoker, No of CHD	1	14	15	11
Multivariable-adjusted HR (95% CI)^a^	NC	1 (reference)	1.10 (0.51–2.38)	0.76 (0.32–1.77)
Current smoker, No of CHD	14	45	32	29
Multivariable-adjusted HR (95% CI)^a^	3.21 (1.68–6.11)***	1 (reference)	1.29 (0.78–2.14)	1.02 (0.59–1.75)

Data above are presented in numbers, HR, and HR (95% CI).

*HR* hazard ratio, *CI* confidence interval, *NSAIDs* non-steroidal anti-inflammatory-drugs, *NC* not calculable.

^a^Adjusted for socioeconomic factors (age, sex, residential area, insurance premium), lifestyle (smoking status, alcohol consumption, and physical activity), medical characteristics (fasting serum glucose, total cholesterol, blood pressure, and Charlson comorbidity index), family history (cardiovascular disease), depression, and medication use (aspirin, statin, anti-hypertensive drugs, anti-diabetic drugs, and NSAIDs) except for the variable used for each stratified analysis.

*p < 0.05, **p < 0.01, ***p < 0.001.

To further test whether the prediagnosis BMI-CHD association remained consistent across different cancer survivor subgroups, we categorized the analytic sample based on age at first cancer diagnosis, sex, insurance premium, physical activity, and cigarette smoking (Table 4). When we computed the multivariable-adjusted HRs and 95% CIs using Model 3, cancer survivors with a prediagnosis BMI of < 18.5 kg/m^2^ showed significantly higher CHD risk compared to those with a prediagnosis BMI of 18.5 to 22.9 kg/m^2^. The statistical significance was attenuated among underweight (prediagnosis BMI: < 18.5 kg/m^2^) cancer survivors aged below 60 years, those with upper half of the insurance premium, and those who were non-smokers.

## Discussion

Using data obtained from a large cohort of patients with cancer, we found that prediagnosis low BMI among cancer survivors was associated with increased subsequent CHD risk. Underweight patients had a 97% higher CHD risk compared to patients with normal weight. This study’s findings suggest that the BMI-CHD risk relationship is U-shaped (increased CHD risk was associated with lower BMI, underweight, overweight, and obesity).

Studies that have helped establish obesity as a major risk factor for CHD did not include underweight subjects; furthermore, these studies tended to merge them into normal-weight groups in statistical analysis. Although some adjustments were made for markers of comorbid conditions and other patient characteristics, and these attenuated some of the excess CHD risk for overweight and obese patients, underweight patients experienced increased CHD risk after the adjustment was made and showed about a two-fold higher risk of subsequent CHD development compared to patients with normal weight. Our findings suggested that lower BMI (< 18.5 kg/m^2^) was associated with higher CHD risk compared to a normal BMI of 18.5 to 22.9 kg/m^2^ in overall number of cancer survivors. Moreover, CHD risk was higher in the underweight group compared to the overweight and obese groups; there was an approximately two- to three-fold increase in the HR for CHD risk in lung and gastric cancer survivors (HR = 3.08; 95% CI: 1.16–8.15 and HR = 2.99; 95% CI: 1.31–6.80, respectively).

One important aspect of this study was the high CHD risk among cancer survivors with low prediagnosis BMI. Some studies dealing with non-cancer-affected general populations have produced similar results. While previous studies have investigated the association between underweight and CHD mortality in the general population^17^ or the relationship between underweight and CHD mortality in survivors of one specific type of breast cancer^18^, our analytic sample included cancer survivors with various cancer sites and examined the association between underweight and subsequent CHD risk using routinely collected medical claims data. Therefore, our findings regarding the association between underweight and subsequent CHD risk provide additional evidence that pre-existing underweight issues could be a potential modifiable risk factor for CHD in patients with cancer. Previously proposed causes of CHD in cancer survivors are mostly multifactorial, involving therapeutic exposures related to the cardiovascular system and comorbidities and lifestyle factors that may increase long-term CHD risk^18,19^.

Several mechanisms could explain the higher CHD risk in underweight patients. First, in this study, the subjects with prediagnosis low BMI were physically inactive; this means that not only fatness but also fitness could be reduced. Physical inactivity and low CRF are well recognized risk factors for CHD, and previous studies have suggested that CRF significantly alters the prognostic implications of fatness in patients with CHD^6^. Second, older underweight cancer survivors often suffer from a lack of protein energy nutrition leading to occasional vital exhaustion which might increase pro-thrombotic and cytokine mediated proinflammatory reactions which, in turn, could trigger acute coronary events. Moreover, the groups with low prediagnosis BMI might have found it hard to cope with the acute CHD event, as they were more likely to have lower fat storage and energy reserves compared to those in the group with normal weight^20,21^. Third, the pathophysiology of CHD may follow a fundamentally different process in the case of underweight patients compared with that of overweight or obese patients. Because CHD is largely attributable to the detrimental effects of adiposity and other modifiable risk factors associated with obesity, underweight patients may have an underlying genetic predisposition to CHD, or they may experience different pathophysiologic processes^22^. Our findings suggest that, rather than factors related to obesity, there might be some more harmful risk factors for CHD in underweight patients. Dangas and colleagues reported an inverse relationship between body weight and coronary calcification in patients with coronary artery disease (CAD) using intravascular ultrasonography^23^. Reports have also indicated a higher prevalence of carotid plaque^20^ and a more severe carotid plaque burden^24^ in underweight patients undergoing percutaneous coronary intervention (PCI). These data suggest that, compared to obese patients, underweight patients may have more severe and complex pathologic features in terms of total vasculature including coronary lesions.

Moreover, our results suggested that CHD risk increased significantly in older underweight patients and those who were current smokers; this has been established as an important risk factor for coronary and carotid atherosclerosis^25^. As these groups already have atherosclerosis risk factors, they might be more vulnerable to CHD events compared to their counterparts. An important aspect to consider in this regard is the potential confounding effect of smoking, a well-known cause of CHD. Although our study did attempt to control for this confounder via statistical adjustment, residual confounding may have occurred.

We conducted stratified analyses based on several cancer categories. A similar trend was observed for patients with lung cancer and gastric cancer. A previous large population-based study showed that survivors of lung cancer and gastric cancer experienced an increased CHD risk compared to the general population after adjustments were made for varying risk factors including BMI^17^. Korean cancer statistics showed that more elderly and leaner patients were suffering from lung and gastric cancer^26^ and that they may have been less likely to gain weight and improve fitness levels after the cancer treatment^27^. In line with our study, Yoon et al. found that lung cancer survivors showed a 26% higher risk for CHD compared to non-cancer control subjects in a Korean nationwide study of 20,458 patients^28^, and in their study, BMI in CHD patients among lung cancer survivors was significantly lower than that among non-cancer control subjects. They suggested that therapeutic modalities including radiation and chemotherapy might induce early microvascular changes and late atherosclerosis, which eventually resulted in CHD.

Our findings also identified a U-shaped relationship between CHD and BMI in the category of gastric cancer survivors. Gastric surgery is associated with decreases in metabolically active body mass; thus, it is related to considerable changes in body composition and fitness level, especially in prediagnosis underweight patients^27^. Body composition changes such as loss of lean muscle mass and weight regain with increased adiposity after gastrectomy in underweight cancer survivors might induce the altered metabolic and immune responses leading to chronic inflammation^29,30^. Previous studies reported a decreased risk of CHD and ischemic stroke and favorable metabolic changes resulting from weight loss, mainly in overweight and obese gastric cancer survivors^27^. In our study, after some adjustments for markers of comorbid conditions and other patient characteristics, these adjustments also attenuated some of the excess CHD risk for overweight and obese patients, but underweight patients experienced increased CHD risk after the adjustment was made. Further research to make an inference on the relevant underlying mechanisms in underweight cancer survivors is necessary.

Although we conducted stratified analyses based on several cancer categories, more research into comparisons with previous studies is necessary because of the absence of gender specific cancers such as breast and prostate cancer^18^.

This study had some limitations. First, BMI does not accurately reflect body composition or evaluate skeletal muscle wasting; moreover, even if BMI remains stable, body composition can vary with age, smoking status, diet, physical activity, and many other identified and non-identified variables, especially after surgical treatment. Appropriate indices should be used in future studies in order to gauge approximate body composition and nutritional status. Second, in the National Health Insurance Service-Health Screening Cohort (NHIS-HEALS), all South Korean adults aged 40 and above are screened at least once every two years. However, the possibility of weight loss due to cancer cachexia, which may be an early signal of nutritional disorder and negative prognostic factors, cannot be completely excluded. As we did not have detailed clinical data on cancer stage, we were unable to assess whether being underweight subsequently increased the risk of adverse health status or a major medical condition or vice versa. Third, we were unable to further categorize different first cancer sites due to the research data policy of the NHIS. Therefore, a few cancer types, such as breast cancer and prostate cancer, were not identifiable in the dataset. Previous studies reported increased CHD risk among cancer survivors, especially with regard to overweight-related cancers including breast cancer and prostate cancer. It is necessary to utilize more detailed information for further investigating whether the BMI-CVD association observed among cancer survivors differs based on the first cancer sites. Fourth, although we made adjustments for a number of potential confounders in our analyses, some possible confounding factors may still exist, and these may have contributed to the identified associations in cancer survivors. In this study, the underweight group was physically inactive, but their physical activity, which was measured using leisure time physical activity, could not reflect actual daily physical activity without information on other domains such as occupation, home, or commuting. Information regarding first cancer treatment could not be included, and we could not therefore consider the effects of cancer treatment (e.g., drug-induced cardiotoxicity and radiotherapy) on CHD event probability^31^. Fifth, we did not focus on whether patients experienced increased BMI after cancer diagnosis because our interests focused on assessing prediagnosis risk factors for CHD among cancer survivors; this study’s nature as a retrospective cohort study also played a major role in this regard.

In this nationally representative, population-based cohort study, we analyzed information regarding diverse health conditions, health behaviors, and biological risk factors in order to investigate the CHD outcomes for underweight patients with cancer. Our study suggests that, among patients with cancer, prediagnosis underweight should be assessed in addition to known CHD risk factors.

To prove the causal effect of prediagnosis underweight with regard to CHD, it is necessary to initiate longitudinal studies that combine sufficient instrumental properties to measure body composition, physical activity, and its presumed pathophysiological mechanisms along with adequately powered, prospective studies targeting the implicated mechanisms.

## Materials and methods

### Study subjects

Study subjects were the investigation the association between prediagnosis BMI and CHD based on first cancer site during cohort enrollment. We collected data of 19,826 cancer survivors from the National Health Insurance Service-Health Screening Cohort (NHIS-HEALS)^32^ from January 1, 2004 to December 31, 2009 using International Classification of Diseases (10th revision [ICD-10]) codes from “C00” to “C97” combined with medical claims data recorded for hospital admission. In this retrospective cohort of cancer survivors, those with cancer diagnoses between January 1, 2002 and December 31, 2003 and those with missing prediagnosis BMI data were excluded. Among patients identified from the NHIS-HEALS, we excluded 2278 patients with missing health screening records prior to the cohort enrollment and also excluded 4048 patients who had been diagnosed with cardiovascular disease prior to the follow-up. Finally, a total of 13,500 cancer survivors were included in the analytic sample and followed up for CHD incidence. This study was conducted in accordance with the Declaration of Helsinki, and the study protocol was approved by the Institutional Review Board (IRB) at Seoul National University Hospital (IRB: E-1807-117-960). We were exempted from obtaining patient consent with regard to reviewing medical claims data by IRB at Seoul National University, because the NHIS-HEALS database used in this study was anonymized according to South Korean personal data protection laws.

### Defining the variables

The primary outcome of this study involved CHD (ICD-10 codes: I20-I25) with hospital admission lasting at least 48 h; this, in turn, was followed up from the date of cancer diagnosis until the date of death from any cause or the end of the follow-up period on December 31, 2015. Determining the validity of CHD events using the NHIS-HEALS database has been described elsewhere^33^. Information on prediagnosis BMI was collected from the national health screening dataset in the NHIS-HEALS prior to the cohort enrollment of the cancer survivors. We grouped patients based on the following prediagnosis BMI ranges according to cutoff points relevant to the Asia–Pacific^34^: < 18.5 kg/m^2^, 18.5–22.9 kg/m^2^, 23.0–24.9 kg/m^2^, and ≥ 25.0 kg/m^2^.

### Data collection

We identified each cancer survivor’s death information by linking the NHIS-HEALS database to the death registry of Statistics Korea, which provides the exact death dates of each enrollee to the NHIS. Socioeconomic (age, sex, residential area, and insurance premium), health behavior (cigarette smoking, alcohol consumption, and physical activity), health status (fasting serum glucose, total cholesterol, blood pressure, and family history), and medical (comorbidity and medication use) information were collected from the eligibility, health screening, and medical claims databases in the NHIS-HEALS. The Charlson Comorbidity Index was calculated using the collective medical claims dataset prior to the cancer diagnosis. We identified pre-existing depression using outpatient-visit or hospital-admission records for depression, (ICD-10 codes: F32 and F33). We adopted the European Society of Cardiology Systematic Coronary Risk Evaluation (ESC SCORE) for low risk regions in order to classify the CHD risk among the cancer survivors based on information about age, sex, cigarette smoking, total cholesterol, and blood pressure. A previous study using the NHIS-HEALS database also used the ESC SCORE for low risk regions for middle-aged Korean populations^35^.

### Statistical analysis

The Cox proportional hazards model was used for calculating CHD risk among cancer survivors based on their prediagnosis BMI categories. We first developed a Cox regression model, which was adjusted for socioeconomic variables including age, sex, residential area, and insurance premium (Model 1). Furthermore, we developed the Cox regression model adjusted for lifestyle (cigarette smoking, alcohol consumption, and physical activity) and medical (fasting serum glucose, total cholesterol, systolic blood pressure, and Charlson comorbidity index) characteristics as Model 2. The final Cox regression model included depression and medication use (aspirin, statin, anti-hypertensive drugs, anti-diabetic drugs, and non-steroidal anti-inflammatory drugs) along with all variables included in Model 1 and Model 2. With regard to normal weight (prediagnosis BMI of 18.5 to 22.9 kg/m^2^), we computed the hazard ratio (HR) and 95% confidence interval (95% CI) for CHD in underweight (prediagnosis BMI: < 18.5 kg/m^2^), overweight (prediagnosis BMI: 23.0 to 24.9 kg/m^2^), and obese (prediagnosis BMI: ≥ 25.0 kg/m^2^) categories using the Cox regression models described above. Proportionality assumption of the Cox proportional hazards models were tested graphically using a log–log plot.

To investigate the association between prediagnosis BMI and CHD based on first cancer site during cohort enrollment, we stratified the analyses by using variables included in the Cox proportional hazards regression Model 3 for lung cancer (ICD-10 code: C34), stomach cancer (ICD-10 code: C16), colorectal cancer (ICD-10 codes: C18–C20), and liver cancer (ICD-10 code: C22) survivors. Other types of cancer at first site, excluding thyroid cancer (ICD-10 code: C73), were grouped separately.

We performed subgroup analyses by stratifying the analytic samples based on age (dichotomized as < 60 years and ≥ 60 years), sex (male and female), insurance premium (upper half and lower half), physical activity (< 3 times per week and ≥ 3 times per week), and cigarette smoking (non-smoker, past smoker, and current smoker) using Model 3 for each category of prediagnosis BMI. Statistical significance was two-sided with a cut-off value of *p* < 0.05. Data collection and statistical analyses were conducted using SAS 9.4 (SAS Institute, Cary, NC, USA).
